# Birth Weight, Season of Birth and Postnatal Growth Do Not Predict Levels of Systemic Inflammation in Gambian Adults

**DOI:** 10.1002/ajhb.22413

**Published:** 2013-06-10

**Authors:** Anna A Richards, Anthony J Fulford, Andrew M Prentice, Sophie E Moore

**Affiliations:** 1 Department of Population Health, Medical Research Council (MRC) International Nutrition Group, London School of Hygiene and Tropical MedicineKeppel Street, London, WC1E, 7HT; 2 MRC Keneba, MRC Unit The GambiaBanjul, The Gambia

## Abstract

**Objectives:**

Studies testing whether systemic inflammation might lie on the causal pathway between aberrant fetal and post-natal growth patterns and later cardiovascular disease have been inconclusive, possibly due to the use of single markers of unknown predictive value. We used repeated measures of a comprehensive set of inflammatory markers to investigate the relationship between early life measures and systemic inflammation in an African population.

**Methods:**

Individuals born in three rural villages in The Gambia, and for whom early life measurements were recorded, were traced (*n* = 320). Fasting levels of eight inflammatory markers (C-reactive protein, serum amyloid A, orosomucoid, fibrinogen, α 1-antichymotrypsin, sialic acid, interleukin-6 and neopterin) were measured, and potential confounding factors recorded. The association between early life measurements and systemic inflammation was assessed using regression analysis.

**Results:**

Levels of most markers were unrelated to early growth patterns. In analyses adjusted for age and sex, more rapid growth between birth and 3 months of age was associated with higher levels of fibrinogen, orosomucoid, and sialic acid. These relationships persisted after further adjustment for body mass index but after full adjustment only the association with fibrinogen remained.

**Conclusions:**

This study provides little evidence that size at birth or growth in early infancy determine levels of inflammatory markers in young Gambian adults. Am. J. Hum. Biol. 25:457–464, 2013. © 2013 Wiley Periodicals, Inc.

There is evidence that size at birth and rate of development during early life, in particular low birth weight (Frankel et al., 1996b; Stein et al., 1996; Forsen et al., 1997; Rich-Edwards et al., 1997; Leon et al., 1998) and accelerated early postnatal growth (Horta et al., 2003; Soto et al., 2003; Jarvelin et al., 2004; Adair 2007; Hemachandra et al., 2007; Min et al., 2007), predict increased risk of future cardiovascular disease (CVD) and its associated risk factors. Despite the large body of evidence in this field it is still not understood whether size at birth, the rate of postnatal growth or a combination of small size at birth followed by accelerated growth is most important for determining disease risk (Gillman, 2002). Furthermore, the biological mechanism(s) underpinning the associations between early life environment and later disease risk also remain unclear. Research in the last decade has also shown that raised levels of systemic inflammatory markers predict later morbidity and mortality from CVD (Danesh et al., 1998,2000,2004r).

Previous studies investigating the relationship between early life environment and systemic inflammation have primarily focused on the relationship between birth weight and C-reactive protein (CRP) (Whincup et al., 1996; Cook et al., 2000; Gillum, 2003; Sattar et al., 2004; Raqib et al., 2007) or fibrinogen (Barker et al., 1992; Fall et al., 1995; Martyn et al., 1995; Frankel et al., 1996a; Leger et al., 1997; Cook et al., 1999; Roseboom et al., 2000). These studies, predominately in white Caucasian populations and typically defining systemic inflammatory status using a single measure of one inflammatory marker, have reported conflicting results. The current study widens the evidence base in this field by investigating whether early life variables, including early postnatal growth, predict levels of a wide range of inflammatory markers in Gambian adults. This is the first study to test this hypothesis in an indigenous African population. Furthermore, this study collects duplicate measures of inflammatory markers to assess which marker(s) most reliably characterises chronic systemic inflammation.

## METHODS

### Study population

The study population was all consenting individuals aged 18–30 years born in three rural villages (Keneba, Kantong Kunda, or Manduar) in West Kiang, The Gambia (West Africa) between 1976 and 1987 and for whom birth weight was recorded as part of the United Kingdom Medical Research Council (MRC) Keneba Antenatal Scheme. Details of this scheme and the research setting are available elsewhere (Rayco-Solon et al., 2004). Participants attended a study clinic at one of two MRC stations depending on whether they lived in rural West Kiang (MRC, Keneba) or in the urban centres near the coast (MRC, Fajara). Logistical limitations meant that only potential participants traced to within 90 min drive from either of the two stations were recruited.

Ethical approval for this study was obtained from the London School of Hygiene and Tropical Medicine Ethical Committee and the joint Gambian Government / MRC Unit The Gambia, Ethics Committee. All study participants gave informed written consent before participating.

### Early life measurements

Birth weight (kg) was recorded by the resident paediatrician or midwife to the nearest 10 g and within 72 h of birth. Gestational age was assessed using the score of Dubowitz et al. (1970). Low birth weight (LBW) was defined as <2,500 g. Postnatal weight was measured regularly, at postnatal clinics, to the nearest 10 g using standard equipment; the exact timing of measurements varied by child but date of measurement was recorded for all weights. Growth velocity from birth to 3 months was calculated as the difference between population-derived sex-specific birth weight standard deviation (SD) score and population-derived sex-specific weight at 3 months SD score, where weight at 3 months was defined as the weight nearest to 3 months that was recorded between 2.0 and 4.0 months of age. The weight nearest 12 months that was recorded between 9.6 and 14.4 months of age was used for weight at 1 year. Hungry (wet) season of birth was defined as a birth month between July to December inclusive and harvest (dry) season between January to June inclusive (Moore et al., 1999).

### Systemic inflammatory markers

Blood samples were collected after an overnight fast and then centrifuged for 20 min at 3,000 rpm and 4°C within 1 h of collection and immediately frozen to −80°C. Serum samples were allowed to clot at room temperature, and then centrifuged and processed as described for plasma. Analyses of CRP, serum amyloid A, α 1-antichymotrypsin (ACT), orosomucoid, interleukin-6 (IL-6), sialic acid and neopterin, were performed at MRC Human Nutrition Research, Cambridge, UK. Fibrinogen was measured at Addenbrooke's Hospital Clinical Laboratory, Cambridge, UK.

CRP was measured using a high-sensitivity particle-enhanced turbidimetric immunoassay (Dade Behring, Milton Keynes, UK) on a Dimension ARX Analyzer (Dade Behring, Milton Keynes, UK). The assay has a lower detection level of 1.1 mg/l. Plasma serum amyloid A was measured in duplicate using the enzyme-linked immunosorbent assay (ELISA) principle (Anogen, Mississauga, Canada). Fibrinogen was measured using the Clauss assay. Plasma orosomucoid was measured using an immunoturbidimetric method (Sentinel, Milan, Italy) adapted for use on the Dimension ARX Analyzer (Dade Behring, Milton Keynes, UK). ACT was measured using an immunochemical assay (Dako, Glostrup, Denmark) adapted for use on the Hitachi 912 analyzer (Roche, Welwyn Garden City, UK). IL-6 was measured in duplicate using a high-sensitivity ELISA principle (Diaclone, Besançon, France). Sialic acid was measured by a colorimetric enzyme assay (Roche, Welwyn Garden City, UK) and adapted for use on the Hitachi 912 Analyzer (Roche, Welwyn Garden City, UK). Serum neopterin was measured in duplicate using a competitive enzyme immunoassay principle (BRAHMS Atiengesellschaft, Berlin, Germany). Interassay coefficients of variation were <9.6% for all analyses.

### Potential confounding factors

#### Anthropometry

Weight was measured to the nearest 100 g using an electronic portable scale (Chasmors, UK) and height to the nearest mm using a portable stadiometer (CMS Weighing Equipment Ltd, London, UK). BMI (kg/m^2^) was calculated and categorized using standard cut-offs (WHO, 2000). Waist and hip circumference (cm) were measured to the nearest 0.1 cm. Central obesity was defined as a waist-to-hip ratio ≥0.90 (men) or ≥0.80 (women) (Dobbelsteyn et al., 2001). Whole body composition was measured using dual energy X-ray absorptiometry (DXA) on a Lunar DPX+ (Lunar Corporation, Madison WI).

#### Infectious disease markers

Participants were only enrolled if considered ‘healthy’ at the time of recruitment, based on a screening questionnaire collecting data on recent clinic visits, current medication use, appetite and recent weight loss. Axillary temperature was also recorded. A thick film was prepared from whole blood to look for the presence of malaria parasites. The remaining whole blood was used to measure white blood cell (wbc), lymphocyte, granulocyte and monocyte counts (10^9^/l) and haemoglobin (g/dl) using a Medonic CA 530 Oden 16 Parameter System Haemoglobinometer (Medonic, Stockholm, Sweden).

#### Chronic disease markers

Fasting glucose, total cholesterol, high density lipoprotein (HDL)-cholesterol, triglyceride and leptin levels were measured at MRC Human Nutrition Research, Cambridge, UK. Plasma glucose concentration was measured using an adaptation of the hexokinase-glucose-6-phosphate dehydrogenase method (Dade Behring, Milton Keynes, UK). Impaired fasting glucose (IFG) was defined by a fasting plasma glucose ≥6.1 and ≤6.9 mmol/l and type 2 diabetes by a level ≥7.0 mmol/l (Genuth et al., 2003). Plasma lipids were measured using enzymatic methods on a Dade Behring Dimension (Dade Behring, Milton Keynes, UK). Low density lipoprotein (LDL)-cholesterol was derived using the Friedewald equation (Friedewald et al., 1972). Leptin was measured by ELISA (R&D Systems, Abingdon, UK). Insulin was measured at Addenbrooke's Hospital Clinical Laboratory, Cambridge, UK using a time-resolved fluoroimmunoassay (AutoDELFIA, PerkinElmer Life & Analytical Sciences, Wallac Oy, Turku, Finland). Blood pressure was measured using a fully automatic digital blood pressure monitor (Omron 7051T, Omron Healthcare, IL. Hypertension in adults was defined by a systolic blood pressure (SBP) ≥140 mm Hg and/or a diastolic blood pressure (DBP) ≥ 90 mm Hg (WHO, 1999).

#### Lifestyle measures

Questionnaire data confirmed whether participants were still in full-time education and their smoking status (current smoker, ex-smoker or never smoked). Smoking status was analyzed as ever or never smoked due to the small number of current smokers in the study population. Additional data were collected, by a female fieldworker, on whether women used oral or injectable hormonal contraceptives.

### Data collection protocol

Markers of systemic inflammation and infectious disease status were assessed at two time points [baseline (Day 0) and 2-weeks (Day 14)]. In a random sub-sample of 15 women these levels were further assessed at Day 28 to investigate the reliability of a single measurement to characterise habitual systemic inflammatory status. All other measurements were collected at baseline only. Baseline data were collected between 23 February and 1 June, 2-week data between 9 March and 15 June and 4-week data between 24 March and 30 March, 2006.

## STATISTICAL METHODS

A total of 209 (65.3%) participants had a CRP and 266 (83.1%) an IL-6 measurement below the minimum assay detection level (<1.1 and <0.8 pg/ml, respectively). CRP and IL-6 were therefore analyzed as binary variables (CRP <1.1 vs. ≥ 1.1 mg/l; IL-6 <0.8 vs. ≥ 0.8 pg/ml). Orosomucoid, ACT, sialic acid and neopterin were log_e_ transformed to normality. A log_e_ transformation of serum amyloid A failed to produce a normal distribution and it was necessary to add 100 to each serum amyloid A value and then take the log_e_ transformation to obtain a normal distribution.

The effects of potential confounding factors (listed in Table 1) on levels of inflammatory markers were assessed using logistic regression analysis for CRP and IL-6 and linear regression analysis for all remaining inflammatory markers. Population-derived SD-scores for continuous measures of chronic and infectious disease were used when examining their association with inflammatory markers. This enabled direct comparison between the effects of different measures on levels of inflammatory markers. SD scores were generated from untransformed data for those variables which were normally distributed and from transformed data (log_e_) for those variables which were transformed to produce a normal distribution. All SD scores were sex-specific. SD-scores could not, however, be generated for anthropometric, body composition and leptin measurements as a number of these measures could not be transformed to produce the normal distribution necessary to generate SD scores. The effect of anthropometric, body composition and leptin measurements on levels of inflammatory markers were examined in males and females separately because of the strong sex-differences in these measures observed within the study population.

**TABLE 1:** Characteristics of the study population by sex at baseline

	Males (*n* = 166)		Females (*n* = 154)	
		
	*n*	Median (IQR)/percent (number)	*n*	Median (IQR)/percent (number)
Characteristic				
Age (years)	166	22.7 (20.1, 24.2)	154	22.8 (20.3, 25.2)
Location (percentage living in rural vs. urban area) (%)	166	15.1 (25)	154	31.8 (49)
Early life measurements				
Birth weight (kg)	166	3.10 (3.03, 3.16)	154	2.87 (2.80, 2.93)
Gestational age (weeks)	136	38.9 (38.7, 39.2)	131	38.6 (38.4, 38.9)
Percent low birth weight (%)	166	8.4 (14)	154	17.5 (27)
Weight at one year (kg)	161	8.28 (8.12, 8.44)	147	7.65 (7.48, 7.82)
Change in SD-score from birth to 3 months (SD-score)	143	−0.02 (−0.18, 0.14)	127	−0.15 (−0.32, 0.03)
Percent born in hungry season	166	51.8 (86)	154	53.9 (83)
Adult adiposity				
Body mass index (kg/m^2^)	166	20.1 (18.9, 21.1)	154	21.1 (19.6, 23.3)
Underweight/normal weight/overweight/obese (%)^a^	166	19.3 / 78.9 / 1.2 / 0.6	154	10.4 / 76.0 / 12.3 / 1.3
DXA total fat (kg)^b^	146	4.1 (3.0, 6.0)	138	13.1 (10.5, 18.2)
Waist-to-hip ratio	166	0.76 (0.73, 0.79)	154	0.76 (0.73, 0.80)
Central obesity (%)^c^	166	0.6 (1)	154	15.6 (24)
Chronic disease				
Systolic blood pressure (mm Hg)	166	120.2 (113.7, 127.7)	154	113.3 (108.7, 119.7)
Diastolic blood pressure (mm Hg)	166	68.7 (65.0, 75.3)	154	70.0 (64.7, 74.3)
Hypertension (%)^d^	166	2.4 (4)	154	3.3 (5)
Glucose (mmol/l)	166	4.8 (4.6, 5.0)	154	4.7 (4.5, 4.9)
Insulin (pmol/l)	166	37.5 (27.1, 48.9)	153	44.5 (29.8, 59.9)
Impaired fasting glucose (%)^e^	166	0.6 (1)	154	0.6 (1)
Type 2 diabetes (%)^f^	166	0(0)	154	0.6 (1)
Total cholesterol (mmol/l)	164	3.7 (3.3, 4.2)	152	4.2 (3.6, 4.7)
Triglycerides (mmol/l)	164	0.61 (0.46, 0.80)	152	0.53 (0.42, 0.64)
HDL cholesterol (mmol/l)	164	1.21 (1.06, 1.40)	152	1.42 (1.21, 1.62)
LDL cholesterol (mmol/l)	164	2.2 (1.8, 2.5)	152	2.5 (2.0, 2.9)
Infectious disease				
White blood cell count (10^9^/l)	164	4.5 (3.9, 5.2)	154	4.9 (4.3, 5.5)
Lymphocyte count (10^9^/l)	163	2.0 (1.7, 2.5)	153	2.0 (1.7, 2.3)
Granulocyte count (10^9^/l)	163	2.0 (1.6, 2.5)	153	2.4 (1.9, 3.0)
Monocyte count (10^9^/l)	163	0.4 (0.3, 0.4)	153	0.4 (0.3, 0.4)
Malaria parasitaemia (%)	166	1.2 (2)	154	0 (0)
Haemoglobin (g/dl)	164	15.7 (14.7, 16.8)	154	13.7 (12.8, 14.5)
Lifestyle				
Ever used tobacco regularly (%)	166	29.5 (49)	154	0 (0)
Ever attended school (%)	166	98.8 (164)	154	93.5 (144)
Hormonal contraceptive use (%)		Not applicable	154	3.3 (5)

DXA = dual energy X-ray absorptiometry.

a Underweight = body mass index (BMI) <18.5 kg/m^2^; normal = 18.5–24.9 kg/m^2^; overweight = 25.0–29.9 kg/m^2^; obese = ≥30.0 kg/m^2^.

b DXA measurements were not available for 36 study participants, all of whom were located in the urban coastal areas and were unable to travel to Keneba (where the DXA machine was located) for measurements.

c Central obesity defined as waist circumference ≥90.0 cm (males) or ≥80.0 cm (females).

d Hypertension was defined as a systolic blood pressure ≥ 140 mm Hg and/or a diastolic blood pressure ≥ 90 mm Hg.

e Impaired fasting glucose was defined as a fasting glucose ≥ 6.1 and ≤ 6.9 mmol/l.

f Type 2 diabetes was defined as a fasting glucose ≥ 7.0 mmol/l.

The associations between birth weight, weight at 1 year and postnatal growth with levels of inflammatory markers were analyzed using logistic regression for CRP and IL-6 and linear regression for all remaining inflammatory markers according to the analytical approach recommended by Lucas et al. (1999). This approach uses four separate models. The ‘early model’ relates early size (e.g. birth weight) to later outcome (e.g. adult fibrinogen level). The ‘later model’ relates later size (e.g. BMI) to later outcome. The ‘combined model’ is the early model adjusted for later size. The ‘interaction model’ is the combined model including an early size/later size interaction term (e.g. birth weight*BMI). Associations between low birth weight and season of birth with levels of inflammatory markers were investigated using logistic regression for CRP and IL-6 and linear regression for all remaining inflammatory markers. All analyses were undertaken twice: first, adjusting for age and sex only and second, adjusting for all those potential confounding factors observed to predict levels of each inflammatory marker. Study participants had up to three measures of each inflammatory marker recorded at baseline (Day 0), 14 days and 28 days. Of the 320 participants for whom levels of inflammatory markers were available at Day 0, 303 had repeat measures at Day 14 and a further 15 at Day 28. Intraclass correlation coefficients were used to generate reliability estimates for continuous inflammatory variables using all available data from Day 0, Day 14 and Day 28. In order to get the most accurate measure of systemic inflammation (i.e. a measure that was not ‘falsely’ elevated by underlying infection) the lowest level of each inflammatory marker were used in all analyses. As orosomucoid, ACT, sialic acid, neopterin and serum amyloid A were log_e_ transformed to normality, the β-coefficients generated from their linear regression models are presented as a percentage unit increase; data from untransformed variables (fibrinogen) are presented as absolute changes.

## RESULTS

### Selection of study participants

**Fig. 1:**
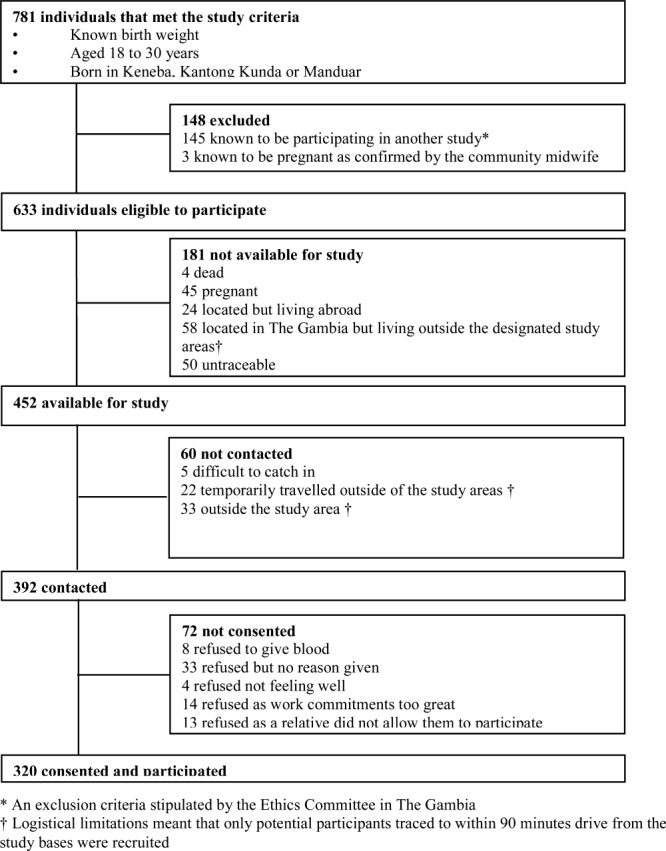
Flow chart summarising the selection of study participants.

Figure 1 describes the selection of study participants and lists reasons for noninclusion. A total of 781 individuals met the study criteria, of whom 148 were excluded prior to tracing. Fieldworkers traced the remaining 633 eligible individuals of whom 181 were subsequently found to be unavailable for study. Fieldworkers were able to contact 86.7% (*n* = 392) of the 452 individuals traced of whom 72 (18.4%) declined to participate. The majority of those who declined were male (n = 46; 63.9%). The remaining 320 individuals represent 70.8% of those traced and available for study (*n* = 452).

Compared with nonparticipants, study participants were younger (mean (95% confidence interval) age 22.2 (21.8, 22.5) vs 23.0 (22.7, 23.3) years; *P* = 0.0001) and a slightly higher percentage were male (51.9 vs 45.3%; *P* = 0.07). Mean birth weight, gestational age, change in SD score from birth to 3 months and weight at 1 year and percentage born during the hungry season or low birth weight were not different between participants and nonparticipants.

## CHARACTERISTICS OF THE STUDY PARTICIPANTS

Table 1 describes the characteristics of the study population by gender. Forty-one participants (12.8%) were born low birth weight with a higher prevalence in female compared with male participants (17.5 vs. 8.4%.; *P* = 0.02). Gestational age ranged from 32.0 to 41.6 weeks with 30 participants (11.2%) born premature (<37 weeks gestation). The majority of participants had a BMI within the normal range although the percentage overweight, or centrally obese, was considerably higher in females compared to males (*P* < 0.001 for both). As expected in young Gambian adults there was a low prevalence of hypertension (<3%), IFG (<1%) and type 2 diabetes (<1%). Less than 1% of the study population had asymptomatic malaria. There was a clear sex-difference in tobacco use with no females, compared with 30% of males reporting ever using tobacco regularly. Few women (3.3%) used hormonal contraceptives.

Table 2 presents the full summary statistics and reliability estimates for each inflammatory marker. There was considerable variation in the reliability estimates between inflammatory markers; the highest estimate (0.718) was observed for sialic acid.

**TABLE 2:** Summary statistics and reliability estimates of inflammatory markers for the total population

Inflammatory marker	*n*	Mean (SD)	Range	*n*	Reliability estimate (SE)	95 % confidence interval of the reliability estimate
Serum amyloid A (ng/ml)^a^	320	1308 (628, 2488)	0.55, 16000	641	0.498 (0.042)	0.416, 0.581
Fibrinogen (g/l)	320	2.8 (0.6)	1.19, 5.61	643	0.405 (0.047)	0.313, 0.496
Orosomucoid (mg/dl)^a^	320	68.8 (59.2, 78.4)	39.0, 157.7	640	0.688 (0.030)	0.630, 0.746
α 1-antichymotrypsin (g/l)^a^	320	0.28 (0.25, 0.30)	0.19, 0.60	643	0.630 (0.034	0.564, 0.696
Sialic acid (mg/dl^a^	320	66.4 (61.2, 72.4)	46.8, 109.6	643	0.718 (0.027)	0.665, 0.771
Neopterin (nmol/l)^a^	320	7.45 (6.40, 8.63)	4.18, 18.25	633	0.532 (0.041)	0.453, 0.612

Inflammatory marker	*n*	Percent (*n*)	Range^b^			
C-reactive protein < 1.1 (mg/l)	320	65.3 % (209)	0.55, 13.6			
Interleukin-6 < 0.8 (pg/ml)	320	83.1 % (266)	0.4, 15.5			

IQR = inter-quartile range; SD = standard deviation.

a Logged data used to report geometric mean (inter-quartile range).

b Range of levels in those individuals categorised as having a C-reactive protein level ≥ 1.1 mg/l and an interleukin-6 level ≥ 0.8 pg/ml.

### Association between early life exposures and systemic inflammation

#### Regression analysis adjusted for age and sex

In regression analyses, each adjusted for age and sex, there was no evidence that birth weight, low birth weight, weight at 1 year or season of birth predicted levels of systemic inflammatory markers. There was evidence that a higher change in SD score from birth to 3 months was associated with higher levels of fibrinogen, orosomucoid and sialic acid but no association was observed with the remaining five markers. For each one unit increase in change in SD score fibrinogen levels increased by 0.11 g/l (95% CI 0.03–0.18 g/l; *P* = 0.004), orosomucoid levels increased by 3% (95% CI 0.4–5.4%; *P* = 0.02) and sialic acid levels by 2% (95% CI 1–4%); *P* = 0.003).

#### Regression analysis fully adjusted for potential confounding factors

For each inflammatory marker, multiple regression analyses were used to adjust for measures of location, chronic disease, infectious disease and lifestyle (as listed in Table 1) that predicted levels of that inflammatory marker. To facilitate comparisons with other studies, and because data were available for all study participants, BMI was used as the primary measure of adult adiposity. After full adjustment there was no association between birth weight, low birth weight, weight at 1 year or season of birth and levels of inflammatory markers. After full adjustment, early postnatal growth still predicted higher levels of adult fibrinogen (0.10 (95% CI 0.03–0.16) g/l; *P* = 0.007) and showed a weak positive association with sialic acid (1.00 (95%-0.05 to 2.92)%; *P* = 0.06) but any association with orosomucoid levels was removed. Table 3 reports the relationship between early postnatal growth and fibrinogen according to the analytical approach recommended by Lucas and colleagues. Supporting Information Tables S1-A–D and S2-A–D present the regression analyses for four selected markers (CRP, orosomucoid, sialic acid and IL-6) by birth weight and early postnatal growth.

**TABLE 3:** Linear regression associations between postnatal growth (change in standard deviation (SD)-score from birth to three months) and adult levels of fibrinogen?

		Postnatal growth (change in SD-score from birth to 3 months)		Body mass index (kg/m^2^)		Interaction^b^	
				
Regression model^a^	*n*	β-coefficient (95 % CI)	*P*-value	β-coefficient (95 % CI)	*P*-value	β-coefficient (95 % CI)	*P*-value
(A) Adjusted for age and sex							
Early	270	0.11 (0.03, 0.18)	0.004				
Later	270			0.04 (0.01, 0.07)	0.004		
Combined	270	0.10 (0.03, 0.17)	0.006	0.04 (0.01, 0.07)	0.01		
Interaction^b^	270					0.01 (−0.02, 0.04)	0.42
(B) Adjusted for age, sex and all factors which predicted fibrinogen levels^c^							
Early	263	0.10 (0.03, 0.16)	0.007				
Later	263			0.04 (0.01, 0.06)	0.01		
Combined	263	0.09 (0.02, 0.16)	0.008	0.04 (0.01, 0.06)	0.01		
Interaction^b^	263					0.01 (−0.01, 0.04)	0.40

β-coefficient [95% confidence interval (CI)] were calculated using linear regression and represent the increase in fibrinogen for each one unit increase in the explanatory variable.

a The early model relates early size (postnatal growth) to later outcome (adult fibrinogen). The later model relates later size (adult body mass index) to later outcome (adult fibrinogen). The combined model is the early model adjusted for later size (adult body mass index).

b The interaction model is the combined model including an early size/later size interaction term (postnatal growth × body mass index).

c Further adjusted for HDL-cholesterol, white blood cell, granulocyte and monocyte counts, malaria parasitaemia and tobacco use.

There was no evidence that adjusting for DXA measures of adiposity altered the association between postnatal growth and levels of inflammatory markers (data not shown). The only exception to this was that adjustment for DXA, total or percent fat, rather than BMI, forced the association between postnatal growth and levels of sialic acid toward the null.

## DISCUSSION

The current study aimed to test the hypothesis that systemic inflammation is young adulthood is predicted by early life exposures. Using a comprehensive set of markers in a cohort of 320 young Gambian adults, we have found little evidence for an effect of a number of early life parameters on later inflammatory status. This is the first published study to investigate this association in an indigenous African population. The inclusion of a range of early life measures, in particular early postnatal growth, and a large and varied number of markers to assess systemic inflammation considerably widens the evidence base in this field.

To date, the only published data supporting an association between prenatal exposures and systemic inflammation comes from studies looking at the relationship between birth weight and either CRP or fibrinogen. Previous studies in infants and young children have reported no association between birth weight and levels of CRP (Cook et al., 2000; Gillum, 2003; Oldroyd et al., 2009), although one study was hampered by the failure to use a high-sensitivity assay (Gillum, 2003). Studies in adults, including data from 5,849 Finnish men and women aged 31 years (northern Finland 1966 Birth Cohort; Tzoulaki et al., 2008) and 1,603 middle-aged Scottish adults (Sattar et al., 2004) have reported that, after adjustment for confounding factors, lower birth weight was associated with higher levels of CRP. A separate analysis of the northern Finland Birth Cohort also reported that lower birth weight predicted higher adult total leukocyte count (Canoy et al., 2009). Likewise, data from the Philippines shows that birth weight was negatively associated with CRP in adulthood (McDade et al., [Bibr b33],[Bibr b34]), A number of previous studies have investigated the association between birth weight and fibrinogen (as a measure of CVD risk). The findings from previously published studies in adults are inconsistent (Martyn et al., 1995; Frankel et al., 1996a) but in line with this study, the majority have reported no association (Barker et al., 1992; Leger et al., 1997; Roseboom et al., 2000).

Few studies have investigated the association between postnatal growth and systemic inflammation. Data from 3,827 adults in the 1982 Pelotas (Brazil) birth cohort suggest that rapid weight gain across the life course predicts higher CRP at age 23 years (Nazmi et al., 2009). In the northern Finland birth cohort, participants with highest tertile body mass index (BMI) at 31 years and lowest tertile birth weight had the highest average CRP levels (Tzoulaki et al., 2008). It has been suggested that the association between low birth weight and increased CVD risk factors in later life may not be a result of low birth weight per se but the subsequent rapid postnatal growth (Kuh and Ben-Shlomo, 1997). The potential mechanisms explaining any association between rapid postnatal growth and adult systemic inflammation are not understood and cannot be tested by the current study design. A series of systematic reviews have reported that rapid catch-up growth is a risk factor for later obesity (Baird et al., 2005; Monteiro and Victora, 2005; Ong and Loos, 2006). One potential mechanism, therefore, is that later adiposity determined by catch-up growth explains any association between rapid postnatal growth and levels of inflammatory markers. In the current study, adjustment for BMI did not alter the association between postnatal growth and fibrinogen; suggesting that in this study population catch-up growth, independent of adult adiposity, was important. A second potential mechanism is that rapid catch-up growth and raised levels of fibrinogen and sialic acid arise from a common genotype. However, there is currently no evidence in support of this hypothesis, and it cannot be tested from the data collected in the current study.

A number of published reports have tested the repeatability of CRP within individuals across longitudinal collections (e.g., Macy et al., 1997; McDade et al., 2010; Ockene et al., 2001; McDade et al., 2012). However, only one previous study has collected repeat measures of a range of inflammatory markers to assess which most reliably characterizes systemic inflammation (Browning et al., 2004). Browning et al. (2004) compared three repeat measures of a range of cytokines and acute phase response markers (including CRP) in 15 overweight white Caucasian UK women over a 6 month period and concluded that IL-6 and sialic acid were best at characterising systemic inflammation with a single measure. In the current study CRP and IL-6 were analyzed as binary variables and the repeatability of both measures could not be assessed. However, of the six remaining inflammatory markers studied sialic acid had the highest repeatability co-efficient. The observation, in two different populations, that a single measure of sialic acid provides a reliable measure of habitual systemic inflammation gives further weight to the conclusion by Browning et al., that future studies should consider using sialic acid as a measure of systemic inflammation. Sialic acid is not an acute phase protein but the terminal glycoprotein found in a number of acute phase proteins. It has been estimated that these glycoproteins account for approximately 70% of the total sialic acid concentration (Lindberg et al., 1993). Browning et al. argue that sialic acid may therefore provide an “integrated measure of the inflammatory response” which is “less prone to the day-to-day variability of individual markers”; there is no reason why this explanation is not pertinent to the current (and other) study populations.

The primary strength of the current study is the collection of data on eight inflammatory markers allowing a more detailed investigation into the association between early life parameters and systemic inflammation than previous studies. Furthermore, the collection of repeat samples of inflammatory markers enabled the reliability of a single measure to be assessed for the first time in an indigenous African population. The very low incidence of risk factors for CVD in this population limited the possibility that systemic inflammation was elevated due to clinical or subclinical atherosclerosis.

One of the difficulties of measuring systemic inflammation in an African, compared with UK, population is that levels of markers may be elevated as a result of infectious disease. Levels of infectious disease in the study population were limited by carrying out data collection in the dry season (November to May) [the rains from June to October coincide with an increase in disease transmission (Brewster and Greenwood, 1993)]. The prevalence of malaria parasitaemia and incidence of elevated levels of inflammatory markers indicated that the population under investigation were healthy at the time of study (for example, only 2% of individuals had CRP levels > 6 mg/dl).

The majority of published data linking inflammation to later CVD comes from industrialised settings. The possibility exists, therefore, that within rural African or other indigenous populations inflammation may not always be a risk factor for CVD. In the Tsimane tribe from Bolivia, for example, markers of infection and inflammation were much higher than among comparable US adults (Gurven et al., 2009). Such data suggest that inflammation in such settings may be offset by other factors such as an active lifestyle and favorable body mass, and hence not result in morbidity or mortality from chronic degenerative diseases.

A critical weakness of the study is that the population measured represents only 41% of those who met the initial study criteria (*n* = 781). Comparisons between participants and nonparticipants however suggest no differences between the two groups in the exposure variables available for analysis. In addition, it is important to highlight that this study was investigating comparisons within-subjects and it is unlikely that in nonparticipants any associations between early life factors and levels of inflammatory markers would be in the opposite direction to that observed in participants.

It has been suggested that the association between birth weight and other determinants of cardiovascular disease (e.g. blood pressure) amplifies with age; the relatively young age of the study population (19–30 years) may have prevented any association between early life events and levels of inflammatory markers being observed. Furthermore, the study participants were relatively lean and therefore underlying associations may exist but remain hidden.

A technical limitation of the study design was that despite using high sensitivity kits the CRP and IL-6 assays were not sufficiently sensitive in the lower ranges. In total, 65 and 83% of participants had levels of CRP and IL-6 below the assay ranges, respectively; CRP and IL-6 were subsequently analyzed as binary variables which limited the power to observe any association with early life factors. No evidence-base was available with which to decide the cut-offs for generating the binary variables. It is possible that the cut-offs used in this study (above and below the lowest assay cut-off) also limited observations on the effect of early life factors on levels of CRP and IL-6.

## CONCLUSIONS

The main hypothesis of this study was that early life programming of systemic inflammation was a mechanism to explain the association between poor early life growth and increased risk of adult CVD; this study provided little evidence to support this hypothesis among young, lean Gambians.
