# Supplementary material for: Birth Weight, Season of Birth and Postnatal Growth Do Not Predict Levels of Systemic Inflammation in Gambian Adults
Source: Am J Hum Biol. 2013 Jun 10;25(4):457–64. doi: 10.1002/ajhb.22413 (PMC3736150; doi:10.1002/ajhb.22413)
Supplement: Supplementary file 1 [file ajhb0025-0457-SD1.doc]

**Tables S1 A-D. Associations between birth weight and adult levels of selected inflammatory markers.**

**Table S1-A** C-reactive protein (ng/mL).

**Table S1-B** Orosomucoid (mg/dL).

**Table S1-C** Interleukin-6 (pg/mL).

**Table S1-D** Sialic Acid (mg/dL).

**Tables S2 A-D. Associations between postnatal growth (change in standard deviation (SD)-score from birth to 3 months) and adult levels of selected inflammatory markers.**

**Table S2-A** C-reactive protein (ng/mL).

**Table S2-B** Orosomucoid (mg/dL).

**Table S2-C** Interleukin-6 (pg/mL).

**Table S2-D** Sialic Acid (mg/dL).

**Table S1-A** Logistic regression associations between birth weight (kg) and adult levels of C-reactive protein (ng/mL).

|  |  | **Birth weight (kg)** | | **Body mass index (kg/m^2^)** | | **Interaction †** | |
| --- | --- | --- | --- | --- | --- | --- | --- |
| **Regression model *** | **N** | **Odds Ratio (95 % CI)** | **p-value** | **Odds Ratio (95 % CI)** | **p-value** | **Odds Ratio (95 % CI)** | **p-value** |
| **(A) Adjusted for age and sex** | | | | | | | |
| **Early** | 320 | 0.87 (0.50, 1.54) | 0.64 |  |  |  |  |
| **Later** | 320 |  |  | 1.21 (1.10, 1.34) | <0.001 |  |  |
| **Combined** | 320 | 0.76 (0.42, 1.36) | 0.34 | 1.22 (1.10, 1.35) | <0.001 |  |  |
| **Interaction** | 320 |  |  |  |  | 0.85 (-.67, 1.09) | 0.20 |
| **(B) Adjusted for age, sex and all factors which predicted C-reactive protein levels ‡** | | | | | | | |
| **Early** | 312 | 0.99 (0.55, 1.80) | 0.98 |  |  |  |  |
| **Later** | 312 |  |  | 1.19 (1.07, 1.32) | 0.001 |  |  |
| **Combined** | 312 | 0.86 (0.47, 1.57) | 0.62 | 1.19 (1.07, 1.33) | 0.001 |  |  |
| **Interaction** | 312 |  |  |  |  | 0.84 (0.65, 1.08) | 0.17 |

Odds ratios (95 % confidence intervals (CI)) were calculated using logistic regression and represent the odds of having a C-reactive protein (CRP) ≥ 1.1 mg/L for each one unit increase in the explanatory variable.

* The early model relates early size (birth weight) to later outcome (adult CRP).

The later model relates later size (adult body mass index) to later outcome (adult CRP).

The combined model is the early model adjusted for later size (adult body mass index).

† The interaction model is the combined model including an early size/later size interaction term (birth weight*body mass index).

‡ Further adjusted for HDL-cholesterol and granulocyte count.

**Table S1-B** Linear regression associations between birth weight (kg) and adult levels of orosomucoid (mg/dL).

|  |  | **Birth weight (kg)** | | **Body mass index (kg/m^2^)** | | **Interaction †** | |
| --- | --- | --- | --- | --- | --- | --- | --- |
| **Regression model *** | **N** | **β-coefficient (95 % CI)** | **p-value** | **β-coefficient (95 % CI)** | **p-value** | **β-coefficient (95 % CI)** | **p-value** |
| **(A) Adjusted for age and sex** | | | | | | | |
| **Early** | 320 | -0.02 (-0.08, 0.04) | 0.49 |  |  |  |  |
| **Later** | 320 |  |  | 0.02 (0.01, 0.03) | <0.001 |  |  |
| **Combined** | 320 | -0.03 (-0.09, 0.02) | 0.27 | 0.02 (0.01, 0.03) | <0.001 |  |  |
| **Interaction** | 320 |  |  |  |  | -0.01 (-0.03, 0.01) | 0.56 |
| **(B) Adjusted for age, sex and all factors which predicted orosomucoid levels ‡** | | | | | | | |
| **Early** | 313 | 0.02 (-0.04, 0.07) | 0.54 |  |  |  |  |
| **Later** | 313 |  |  | 0.01 (0.01, 0.02) | 0.001 |  |  |
| **Combined** | 313 | 0.01 (-0.05, 0.06) | 0.84 | 0.01 (0.006, 0.023) | 0.001 |  |  |
| **Interaction** | 313 |  |  |  |  | -0.01 (-0.03, 0.01) | 0.54 |

β-coefficient (95 % confidence interval (CI)) were calculated using linear regression and represent the increase in orosmucoid for each one unit increase in the explanatory variable.

* The early model relates early size (birth weight) to later outcome (adult orosmucoid).

The later model relates later size (adult body mass index) to later outcome (adult orosmucoid).

The combined model is the early model adjusted for later size (adult body mass index).

† The interaction model is the combined model including an early size/later size interaction term (birth weight*body mass index).

**‡** Further adjusted for HDL-cholesterol and granulocyte count.

**Table S1-C** Logistic regression associations between birth weight (kg) and adult levels of Interleukin-6 (pg/mL).

|  |  | **Birth weight (kg)** | | **Body mass index (kg/m^2^)** | | **Interaction †** | |
| --- | --- | --- | --- | --- | --- | --- | --- |
| **Regression model *** | **N** | **Odds Ratio (95 % CI)** | **p-value** | **Odds Ratio (95 % CI)** | **p-value** | **Odds Ratio (95 % CI)** | **p-value** |
| **(A) Adjusted for age and sex** | | | | | | | |
| **Early** | 320 | 0.93 (0.46, 1.90) | 0.85 |  |  |  |  |
| **Later** | 320 |  |  | 0.98 (0.87, 1.11) | 0.77 |  |  |
| **Combined** | 320 | 0.94 (0.46, 1.92) | 0.88 | 0.98 (0.87, 1.11) | 0.78 |  |  |
| **Interaction** | 320 |  |  |  |  | 0.93 (0.70, 1.23) | 0.61 |
| **(B) Adjusted for age, sex and all factors which predicted Interleukin-6 levels ‡** | | | | | | | |
| **Early** | 313 | 0.98 (0.46, 2.10) | 0.97 |  |  |  |  |
| **Later** | 313 |  |  | 0.94 (0.83. 1.06) | 0.31 |  |  |
| **Combined** | 313 | 1.04 (-0.48, 2.23) | 0.93 | 0.94 (0.83, 1.06) | 0.30 |  |  |
| **Interaction** | 313 |  |  |  |  | 0.92 (0.69, 1.22) | 0.55 |

Odds ratio (95 % confidence interval (CI)) were calculated using logistic regression and represent the odds of having an Interleukin-6 (IL-6) level ≥ 0.8 pg/mL for each one unit increase in the explanatory variable.

* The early model relates early size (birth weight) to later outcome (adult IL-6).

The later model relates later size (adult body mass index) to later outcome (adult IL-6).

The combined model is the early model adjusted for later size (adult body mass index).

† The interaction model is the combined model including an early size/later size interaction term (birth weight*body mass index).

‡ Further adjusted for location, HDL-cholesterol and white blood cell, granulocyte and monocyte counts.

**Table S1-D** Linear regression associations between birth weight (kg) and adult levels of Sialic Acid (mg/dL).

|  |  | **Birth weight (kg)** | | **Body mass index (kg/m^2^)** | | **Interaction †** | |
| --- | --- | --- | --- | --- | --- | --- | --- |
| **Regression model *** | **N** | **β-coefficient (95 % CI)** | **p-value** | **β-coefficient (95 % CI)** | **p-value** | **β-coefficient (95 % CI)** | **p-value** |
| **(A) Adjusted for age and sex** | | | | | | | |
| **Early** | 320 | -0.03 (-0.06, 0.01) | 0.13 |  |  |  |  |
| **Later** | 320 |  |  | 0.01 (0.002, 0.012) | 0.006 |  |  |
| **Combined** | 320 | -0.03 (-0.063, 0.002) | 0.07 | 0.01 (0.003, 0.013) | 0.004 |  |  |
| **Interaction** | 320 |  |  |  |  | -0.01 (-0.023, 0.002) | 0.11 |
| **(B) Adjusted for age, sex and all factors which predicted Sialic Acid levels ‡** | | | | | | | |
| **Early** | 311 | 0.002 (-0.03, 0.03) | 0.90 |  |  |  |  |
| **Later** | 311 |  |  | 0.004 (-0.002, 0.009) | 0.17 |  |  |
| **Combined** | 311 | -0.002 (-0.03, 0.03) | 0.91 | 0.004 (-0.002, 0.009) | 0.17 |  |  |
| **Interaction** | 311 |  |  |  |  | -0.01 (-0.021, 0.003) | 0.14 |

β-coefficient (95 % confidence interval (CI)) were calculated using linear regression and represent the increase in Sialic Acid (SA) for each one unit increase in the explanatory variable.

* The early model relates early size (birth weight) to later outcome (adult SA).

The later model relates later size (adult body mass index) to later outcome (adult SA).

The combined model is the early model adjusted for later size (adult body mass index).

† The interaction model is the combined model including an early size/later size interaction term (birth weight*body mass index).

‡ Further adjusted for diastolic blood pressure, insulin, glucose, total cholesterol, triglycerides, LDL-cholesterol and white blood cell, granulocyte and monocyte counts.

**Table S2-A** Logistic regression associations between postnatal growth (change in standard deviation (SD)-score from birth to 3 months) and adult levels of C-reactive protein (ng/mL).

|  |  | **Postnatal growth (change in SD-score from birth to 3 months)** | | **Body mass index (kg/m^2^)** | | **Interaction †** | |
| --- | --- | --- | --- | --- | --- | --- | --- |
| **Regression model *** | **N** | **Odds Ratio (95 % CI)** | **p-value** | **Odds Ratio (95 % CI)** | **p-value** | **Odds Ratio (95 % CI)** | **p-value** |
| **(A) Adjusted for age and sex** | | | | | | | |
| **Early** | 270 | 1.04 (0.80, 1.34) | 0.77 |  |  |  |  |
| **Later** | 270 |  |  | 1.26 (1.09, 1.36) | 0.001 |  |  |
| **Combined** | 270 | 1.01 (0.78, 1.31) | 0.94 | 1.22 (1.09, 1.36) | 0.001 |  |  |
| **Interaction** | 270 |  |  |  |  | 1.04 (0.94, 1.18) | 0.44 |
| **(B) Adjusted for age, sex and all factors which predicted C-reactive protein levels ‡** | | | | | | | |
| **Early** | 265 | 1.02 (0.78, 1.34) | 0.89 |  |  |  |  |
| **Later** | 265 |  |  | 1.21 (1.07, 1.36) | 0.002 |  |  |
| **Combined** | 265 | 1.01 (0.76, 1.33) | 0.96 | 1.21 (1.07, 1.36) | 0.002 |  |  |
| **Interaction** | 265 |  |  |  |  | 1.07 (0.95, 1.19) | 0.27 |

Odds ratio (95 % confidence interval (CI)) were calculated using logistic regression and represent the odds of having a C-reactive protein (CRP) ≥ 1.1 mg/L for each one unit increase in the explanatory variable.

* The early model relates early size (postnatal growth) to later outcome (adult CRP).

The later model relates later size (adult body mass index) to later outcome (adult CRP).

The combined model is the early model adjusted for later size (adult body mass index).

† The interaction model is the combined model including an early size/later size interaction term (postnatal growth*body mass index).

**‡** Further adjusted for HDL-cholesterol and granulocyte count.

**Table S2-B Linear regression associations between postnatal growth (change in standard deviation (SD)-score from birth to 3 months) and adult levels of orosomucoid (mg/dL).**

|  |  | **Postnatal growth (change in SD-score from birth to 3 months)** | | **Body mass index (kg/m^2^)** | | **Interaction †** | |
| --- | --- | --- | --- | --- | --- | --- | --- |
| **Regression model *** | **N** | **β-coefficient (95 % CI)** | **p-value** | **β-coefficient (95 % CI)** | **p-value** | **β-coefficient (95 % CI)** | **p-value** |
| **(A) Adjusted for age and sex** | | | | | | | |
| **Early** | 270 | 0.03 (0.004, 0.054) | 0.02 |  |  |  |  |
| **Later** | 270 |  |  | 0.02 (0.01, 0.03) | <0.001 |  |  |
| **Combined** | 270 | 0.03 (0.002, 0.050) | 0.03 | 0.02 (0.01, 0.03) | 0.001 |  |  |
| **Interaction** | 270 |  |  |  |  | -0.002 (-0.01, 0.01) | 0.74 |
| **(B) Adjusted for age, sex and all factors which predicted orosomucoid levels ‡** | | | | | | | |
| **Early** | 265 | 0.01 (-0.01, 0.04) | 0.28 |  |  |  |  |
| **Later** | 265 |  |  | 0.02 (0.01, 0.02) | 0.002 |  |  |
| **Combined** | 265 | 0.01 (-0.01, 0.04) | 0.32 | 0.01 (0.005, 0.025) | 0.002 |  |  |
| **Interaction** | 265 |  |  |  |  | 0.0001 (-0.01, 0.01) | 0.98 |

β-coefficient (95 % confidence interval (CI)) were calculated using linear regression and represent the increase in orosomucoid for each one unit increase in the explanatory variable.

* The early model relates early size (postnatal growth) to later outcome (adult orosmucoid).

The later model relates later size (adult body mass index) to later outcome (adult orosmucoid).

The combined model is the early model adjusted for later size (adult body mass index).

† The interaction model is the combined model including an early size/later size interaction term (postnatal growth*body mass index).

**‡** Further adjusted for HDL-cholesterol and granulocyte count.

**Table S2-C** Logistic regression associations between postnatal growth (change in standard deviation (SD)-score from birth to 3 months) and adult levels of Interleukin-6 (pg/mL).

|  |  | **Postnatal growth (change in SD-score from birth to 3 months)** | | **Body mass index (kg/m^2^)** | | **Interaction †** | |
| --- | --- | --- | --- | --- | --- | --- | --- |
| **Regression model *** | **N** | **Odds Ratio (95 % CI)** | **p-value** | **Odds Ratio (95 % CI)** | **p-value** | **Odds Ratio (95 % CI)** | **p-value** |
| **(A) Adjusted for age and sex** | | | | | | | |
| **Early** | 270 | 1.13 (0.82, 1.55) | 0.46 |  |  |  |  |
| **Later** | 270 |  |  | 1.00 (0.88, 1.14) | 0.96 |  |  |
| **Combined** | 270 | 1.13 (0.82, 1.55) | 0.46 | 0.99 (0.87, 1.13) | 0.92 |  |  |
| **Interaction** | 270 |  |  |  |  | 1.15 (1.01, 1.31) | 0.03 |
| **(B) Adjusted for age, sex and all factors which predicted Interleukin-6 levels ‡** | | | | | | | |
| **Early** | 266 | 1.04 (0.74, 1.45) | 0.83 |  |  |  |  |
| **Later** | 266 |  |  | 0.95 (0.83, 1.08) | 0.44 |  |  |
| **Combined** | 266 | 1.05 (0.75, 1.47) | 0.80 | 0.95 (0.83, 1.08) | 0.44 |  |  |
| **Interaction** | 266 |  |  |  |  | 1.11 (0.98, 1.27) | 0.10 |

Odds ratio (95 % confidence interval (CI)) were calculated using logistic regression and represent the odds of having an Interleukin-6 (IL-6) level ≥ 0.8 pg/mL for each one unit increase in the explanatory variable.

* The early model relates early size (postnatal growth) to later outcome (adult IL-6).

The later model relates later size (adult body mass index) to later outcome (adult IL-6).

The combined model is the early model adjusted for later size (adult body mass index).

† The interaction model is the combined model including an early size/later size interaction term (postnatal growth*body mass index).

**‡** Further adjusted for HDL-cholesterol and white blood cell, granulocyte and monocyte counts.

**Table S2-D** Linear regression associations between postnatal growth (change in standard deviation (SD)-score from birth to 3 months) and adult levels of Sialic Acid (mg/dL).

|  |  | **Postnatal growth (change in SD-score from birth to 3 months)** | | **Body mass index (kg/m^2^)** | | **Interaction †** | |
| --- | --- | --- | --- | --- | --- | --- | --- |
| **Regression model *** | **N** | **β-coefficient (95 % CI)** | **p-value** | **β-coefficient (95 % CI)** | **p-value** | **β-coefficient (95 % CI)** | **p-value** |
| **(A) Adjusted for age and sex** | | | | | | | |
| **Early** | 270 | 0.02 (0.01, 0.04) | 0.003 |  |  |  |  |
| **Later** | 270 |  |  | 0.01 (0.004, 0.02) | 0.001 |  |  |
| **Combined** | 270 | 0.02 (0.01, 0.03) | 0.005 | 0.01 (0.004, 0.016) | 0.001 |  |  |
| **Interaction** | 270 |  |  |  |  | 0.003 (-0.002, 0.008) | 0.29 |
| **(B) Adjusted for age, sex and all factors which predicted Sialic Acid levels ‡** | | | | | | | |
| **Early** | 264 | 0.01 (-0.0002, 0.0291) | 0.05 |  |  |  |  |
| **Later** | 264 |  |  | 0.01 (0.0004, 0.0125) | 0.04 |  |  |
| **Combined** | 264 | 0.01 (-0.0003, 0.0288) | 0.06 | 0.01 (0.0004, 0.0124) | 0.04 |  |  |
| **Interaction** | 264 |  |  |  |  | 0.003 (-0.002, 0.008) | 0.28 |

β-coefficient (95 % confidence interval (CI)) were calculated using linear regression and represent the increase in Sialic Acid (SA) for each one unit increase in the explanatory variable.

* The early model relates early size (postnatal growth) to later outcome (adult SA).

The later model relates later size (adult body mass index) to later outcome (adult SA).

The combined model is the early model adjusted for later size (adult body mass index).

† The interaction model is the combined model including an early size/later size interaction term (postnatal growth*body mass index).

**‡** Further adjusted for diastolic blood pressure, insulin, glucose, total cholesterol, triglycerides, LDL-cholesterol and white blood cell, granulocyte and monocyte counts.
